# Perovskite Lanthanum‐Doped Barium Stannate: A Refractory Near‐Zero‐Index Material for High‐Temperature Energy Harvesting Systems

**DOI:** 10.1002/advs.202302410

**Published:** 2023-11-23

**Authors:** Hyebi Kim, Geunpil Kim, Young‐Uk Jeon, Wonjun Lee, Byeong‐Hyeon Lee, In Soo Kim, Kwanil Lee, Soo Jin Kim, Jongbum Kim

**Affiliations:** ^1^ Nanophotonics Research Center Korea Institute of Science and Technology (KIST) Seoul 02792 Republic of Korea; ^2^ School of Electrical Engineering Korea University Seoul 02841 Republic of Korea; ^3^ Advanced Analysis Center Korea Institute of Science and Technology (KIST) Seoul 02792 Republic of Korea; ^4^ KIST‐SKKU Carbon‐Neutral Research Center Sungkyunkwan University (SKKU) Suwon 16419 Republic of Korea

**Keywords:** epsilon near zero, lanthanum‐doped barium stannate, near‐zero index, perovskites, refractory materials

## Abstract

The recent interests in bridging intriguing optical phenomena and thermal energy management has led to the demonstration of controlling thermal radiation with epsilon‐near‐zero (ENZ) and the related near‐zero‐index (NZI) optical media. In particular, the manipulation of thermal emission using phononic ENZ and NZI materials has shown promise in mid‐infrared radiative cooling systems operating under low‐temperature environments (below 100 °C). However, the absence of NZI materials capable of withstanding high temperatures has limited the spectral extension of these advanced technologies to the near‐infrared (NIR) regime. Herein, a perovskite conducting oxide, lanthanum‐doped barium stannate (La:BaSnO_3_ [LBSO]), as a refractory NZI material well suited for engineering NIR thermal emission is proposed. This work focuses on the experimental demonstration of superior high‐temperature stability (of at least 1000 °C) of LBSO films in air and its durability under intense UV‐pulsed laser irradiation below peak power of 9 MW cm^−2^. Based on the low optical‐loss in LBSO, a selective narrow‐band thermal emission utilizing a metal‐insulator‐metal (MIM) Fabry–Pérot nanocavity consisting of LBSO films as metallic component is demonstrated. This study shows that LBSO is an ideal candidate as a refractory NZI component for thermal energy conversion operating at high temperatures in air and under strong light irradiations.

## Graphical abstract

Recent research has explored optical phenomena for thermal energy management using near‐zero‐index (NZI) materials. A perovskite oxide, La:BaSnO_3_ (LBSO), as a high‐temperature NZI material for near‐infrared thermal emission is introduced. LBSO exhibits stability at 1000 °C and resistance to intense UV laser. Using LBSO in a metal‐insulator‐metal nanocavity, selective narrow‐band thermal emission, promising for high‐temperature energy conversion, is achieved.

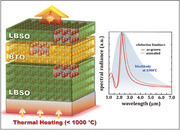

## 1 Introduction

Manipulation of light‐matter interactions relies on the electrodynamic properties of the media, and leads to the exploration of new material platforms with unique optical properties in which the refractive index and/or the permittivity is near zero at a given spectral range.^[^ ^1^, ^2^ ^]^ Recently, thermal emission in epsilon‐near‐zero (ENZ) and the intimately related near‐zero‐index (NZI) media in the form of thin films and metamaterials has received particular attention because of its capability to engineer the directionality and emissivity of thermal emission with the excitation of the ENZ mode.^[^ ^3^, ^4^, ^5^, ^6^, ^7^, ^8^ ^]^ Among various homogeneous ENZ material platforms satisfying the low optical‐loss condition for realizing NZI property,^[^ ^9^ ^]^ phononic materials are widely utilized in radiative cooling systems that require thermal emissions within the atmospheric transparency window of wavelengths between 8 and 13 µm because ENZ wavelengths of these materials are located beyond 6 µm.

To realize the concepts of controlling thermal radiations with NZI materials in the mid‐infrared (MIR) range into energy harvesting systems operating at high temperatures of at least 800 °C, where the near‐infrared (NIR) thermal radiations are dominant, metal oxides are considered as potential candidates. However, the optical properties of metal oxides such as indium tin oxide (ITO)^[^ ^10^, ^11^ ^]^ and doped zinc oxide (ZnO),^[^ ^12^ ^]^ are very sensitive to high temperatures because thermal treatment of such metal oxides can influence their optical properties due to changes in carrier density and mobility.^[^ ^13^ ^]^ Therefore, there is currently no suitable NZI material operational at high temperatures in the NIR spectral range. Although metal nitrides, such as titanium nitride (TiN) and zirconium nitride (ZrN), have been proposed as refractory ENZ materials covering the spectral range from the visible to NIR,^[^ ^14^, ^15^, ^16^, ^17^, ^18^, ^19^, ^20^ ^]^ their high optical losses prevent the realization of NZI characteristics. Moreover, most refractory metals are only able to operate in vacuum due to oxidation in air. Thus, discovering new refractory low‐loss ENZ materials that are chemically stable at high temperatures in air while preserving their optical properties is essential. This is particularly important for realizing conceptual advances in NZI performance for practical application in high‐temperature energy harvesting systems such as thermophotovoltaics (TPV) and solar thermoelectric generators.^[^ ^7^, ^21^, ^22^, ^23^ ^]^

In this paper, we will present experimental results illustrating perovskite lanthanum doped barium stannate (La:BaSnO_3_ [LBSO]) as one of the first candidate for refractory NZI materials operating in the NIR spectral range. Owing to its high electron mobility,^[^ ^24^, ^25^ ^]^ outstanding thermal stability,^[^ ^26^ ^]^ and tunable electrical properties with controlled doping, LBSO has been highlighted as a promising material for transparent electrodes in perovskite solar cells and optoelectronic devices.^[^ ^27^ ^]^ Extensive research has been conducted on utilizing its superior physical properties in the form of thin films,^[^ ^28^, ^29^, ^30^ ^]^ and thus much progress has been made in the synthesis of doped BaSnO_3_ (BSO) films for electronic components. Nevertheless, the optical properties of LBSO films compatible with NZI optical media has been overlooked. In our experiments, we focused on optimizing the optical properties of the LBSO films for low‐loss ENZ, which can serve as NZI components, and uncovering the limitation of the physical properties of the doped BSO as refractory materials. In stark contrast to other refractory materials, crystalline LBSO films possess superior thermal stability up to temperatures of 1000 °C in air without any passivation. Furthermore, the NZI property of LBSO films can be maintained remarkably well under high‐intensity UV‐pulsed laser illumination below average power of 1.8 W cm^−2^. Along with the characterization on high‐temperature stability and intense irradiation durability of LBSO films, we employed LBSO films as refractory metallic component in a metal‐insulator‐metal (MIM) configuration to realize a Fabry–Pérot nanocavity which served as a selective narrow‐band thermal emitter operating in the NIR regime. For the dielectric spacer in MIM nanocavity, a perovskite barium stannate (BaTiO_3_ [BTO]) film was selected due to its small lattice mismatch with LBSO (lattice constant of LBSO: 4.1 Å and BTO: 4.0 Å). From the analysis of lattice strain, we observed that the fully relaxed lattice strain in the MIM structure helps to retain the excellent refractory properties of LBSO materials, resulting in LBSO‐based thermal emitters that can survive under extreme environmental conditions. Our findings reveal the potential of LBSO as refractory NZI materials that can withstand thermal and optical stress for practical applications in thermal management.

## 2 Results and Discussion

**Figure 1:**
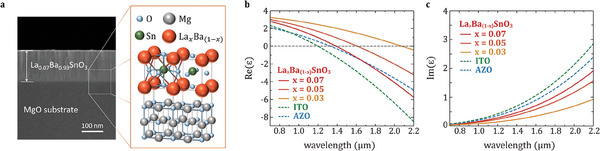
Epsilon‐near‐zero (ENZ) characteristics of LBSO films. a) Cross‐sectional scanning electron microscope (SEM) image and schematic of the crystal structure of LBSO thin film on MgO substrate. b) Real and c) imaginary parts of the dielectric functions of ITO, AZO, and LBSO films. The La doping rate of LBSO film is varied from 3 to 7 wt.%. The ENZ wavelengths of the three materials are at 1.21, 1.34, and 1.44 µm for ITO, AZO, and 7 wt.% La‐doped BSO, respectively.

Highly conductive La‐doped BSO films were deposited on a magnesium oxide (MgO) substrate by pulsed laser deposition (PLD) with a KrF‐excimer laser (*λ* = 248 nm) for source material ablation as shown in **Figure** **1**a. A careful consideration of deposition parameters is required to achieve a low‐loss ENZ property and smooth surface morphology, because certain combinations of deposition temperatures and oxygen partial pressures may induce surface defects such as cracks and voids (see Figure S3, Supporting Information). The doping ratio of lanthanum (La) was varied from 3 to 7 wt.% and all LBSO films were deposited at a substrate temperature of 750 °C and an oxygen partial pressure of 100 mTorr, which are the optimal conditions to achieve the lowest optical loss. We achieved precise stoichiometry for the dopant (*L*a) using commercial targets with 3, 5, and 7 wt.% La. This was subsequently confirmed through Energy‐dispersive X‐ray spectroscopy (see Figure S1, Supporting Information).

For characteristic comparison, an AZO film deposited on MgO substrate via PLD and commercial ITO film on glass substrate (MTI Corp.) were prepared, and the thicknesses of the three materials (ITO, AZO, and LBSO) were kept constant at 170 ± 10 nm. The dielectric functions of all the films were measured with ellipsometry by fitting a lossy Drude oscillator model:

(1) $$\varepsilon \left(\omega \right)=\varepsilon_{\infty }-\frac{\omega_{p}^{2}}{\omega \left(\omega +i\Gamma_{p}\right)}$$

where, ε_∞_ is the high‐frequency limit of the permittivity, ω_p_ is the plasma frequency (proportional to the carrier density), and Γ_p_ is the damping factor (inversely proportional to the carrier mobility). As shown in the dielectric functions (Figure 1b,c), all three material systems exhibit ENZ conditions within the NIR range; 1.21, 1.34, and 1.44 µm for ITO, AZO, and 7 wt.% La‐doped BSO film, respectively, with the imaginary part of the permittivity (*Im*(ε)) at ENZ points of 0.41 (ITO), 0.47 (AZO), and 0.45 (LBSO). The results indicate that the optical properties of the LBSO are suitable to be considered as an alternative NZI material in the NIR range as they satisfy the permittivity condition defined as $Im\left(\varepsilon \right)<\sqrt{1-Re(\varepsilon )}$, where *Re*(ε) and *Im*(ε) are the real and imaginary part of the permittivity, respectively. The ENZ wavelength can be broadly tuned from 1.44 to 2.1 µm by controlling the doping ratio (*x*) of La while maintaining the low‐loss characteristics. The optical properties (ω_p_ and Γ_p_) and the electrical properties including electron mobility (µ_e_) and carrier concentration (*n*_e_) of all materials examined in this experiment is provided in **Table** **1**. We note that the high mobility of LBSO films helps to compensate for its large µ_∞_, which in‐turn satisfies the NZI condition by decreasing the imaginary part of the permittivity at the ENZ wavelength.

**Table 1:** Drude model parameters and electrical properties.

	ε_∞_	ω_**p**_ [*eV*]	Γ_**p**_ [*eV*]	*n*_**e**_ [*cm* ^−3^]	µ_**e**_ [*cm* ^2^ *V* ^−1^ *s* ^−1^]
ITO	4.00	2.05	0.105	2.04E21	23.19
AZO	3.20	1.68	0.135	8.53E20	20.93
La_0.07_Ba_0.93_SnO_3_	4.35	1.81	0.089	5.28E20	85.92
La_0.05_Ba_0.95_SnO_3_	4.35	1.55	0.095	3.75E20	84.63
La_0.03_Ba_0.97_SnO_3_	4.35	1.19	0.09	2.31E20	82.66

The fitting parameters of conducting oxide films with a lossy Drude model consisting of infinite dielectric constant (ε_∞_), plasma frequency (ω_p_), and damping coefficient (Γ_p_).

The electrical properties (carrier density (*n*_e_) and mobility (µ_e_)) obtained by hall measurement.

**Figure 2:**
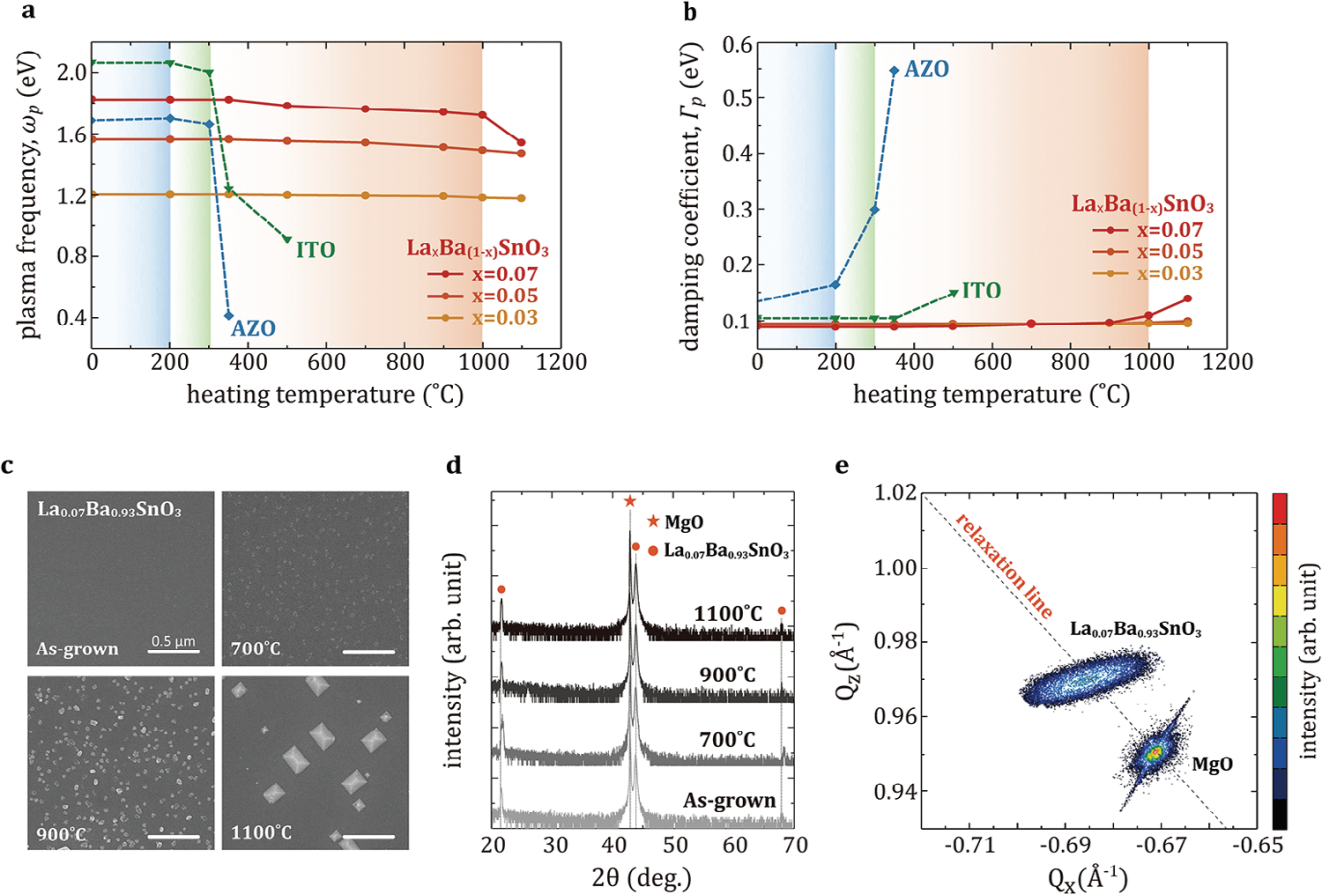
Refractory property of LBSO thin film under air atmosphere. a) Plasma‐frequency (ω_p_) and b) damping coefficient (Γ_p_) of ITO, AZO, and LBSO films with three different La doping rate as a function of the heating temperature. c) SEM images of the surface morphology and d) X‐ray diffraction (XRD) patterns of the LBSO films before and after heating at high temperatures of 700, 900, and 1100 °C. e) The reciprocal space mapping (RSM) of the (224) diffraction of the as‐grown LBSO film deposited on the MgO substrate. Gray dashed line corresponds to the condition of strain relaxation. The diffraction peak position of the LBSO film located on the relaxation line shows that the LBSO film is fully relaxed at the grown stage.

The refractory properties of LBSO films were characterized by monitoring the change of optical properties upon a cycle of heating and cooling in ambient air. For heating, the samples were maintained at designated temperatures (200–1100 °C) for 1 h and for cooling, the samples were cooled down to room temperature at a rate of 5 °C min^−1^ to prevent cracking. The ITO and AZO films were treated in the same way to compare the thermal stability of all conducting oxides examined in this study. The *p*‐polarized transmittances with an incident angle of 60° clearly display the variation of the ENZ wavelength and optical loss from the spectral location and intensity of the absorption dip induced by the ENZ mode (see Figure S2, Supporting Information). To accurately compare the impact of exposure to high temperature, the optical properties (ω_p_ and Γ_p_) of all materials are plotted in **Figure** **2**a,b. Both ITO and AZO maintain their optical properties up to temperatures of 300 °C, while considerable changes in ω_p_ are observed as the temperature approaches 350 °C. In particular, Γ_p_ of the AZO is less stable than that of the ITO even at lower temperatures. It indicates that oxygen defects within the films initially generated to achieve high carrier density actually plays a significant role in degrading the thermal stability in air. On the other hand, ω_p_ and Γ_p_ of the LBSO films remain consistent up to a temperature of 350 °C, with only a marginal change of ≈5% before a noticeable change is observed at 1100 °C. The maximum operating temperature of the LBSO as a refractory material is comparable to that of the existing plasmonic refractory materials such as TiN,^[^ ^18^, ^19^, ^31^ ^]^ molybdenum (Mo),^[^ ^32^, ^33^ ^]^ iridium (Ir),^[^ ^34^ ^]^ and tungsten (W),^[^ ^35^, ^36^, ^37^, ^38^, ^39^ ^]^ which have been reported to be stable up to temperatures of 900, 1000, 1000, and 1200 °C, respectively (see **Table** **2**). The results highlight the fact that optical properties of LBSO films are preserved in air without the aid of a passivating layer (e.g., Al_2_O_3_ or HfO_2_) to prevent oxidation in ambient air, which clearly reveals that the LBSO system is well‐suited for applications with demanding stability requirements, especially the ones designed for high temperature operation in air atmosphere.

**Table 2:** Refractory materials.

Material	Heating Temperature [°C]	Time Duration [h]	Atmosphere	Passivation	Comment
W^[^ ^35^ ^]^	1000	3	Vacuum	–	Metamaterial (Selective Emitter)
W^[^ ^36^ ^]^	1200	10	Vacuum	HfO_2_	Metasurface (Absorber/Emitter)
W^[^ ^37^ ^]^	800/600	4/4	Vacuum/Air	Al_2_O_3_	Multilayer (Absorber)
W^[^ ^38^ ^]^	1400	6	Vacuum	HfO_2_	Metamaterial (Emitter)
W^[^ ^39^ ^]^	1000	24	Ar		Nanodisc (Thermal Emitter)
TiN^[^ ^14^ ^]^	800	8	Vacuum	–	Metamaterial (Absorber)
TiN^[^ ^18^ ^]^	1400	8	Vacuum	–	Thin film
TiN^[^ ^42^ ^]^	600	6	Vacuum	–	Metasurface (Absorber)
TiN^[^ ^43^ ^]^	800	2	Vacuum	–	Metasurface (Absorber)
TiN^[^ ^31^ ^]^	800	8	Nitrogen	Si_3_N_4_	Thin film (Thermal Emitter)
TiN^[^ ^44^ ^]^	100	0.5	Air	–	Nanodisc
Ta^[^ ^45^ ^]^	1000/900	1/144	Argon/Argon	HfO_2_	Photonic Crystal (Thermal Emitter)
Ta^[^ ^46^ ^]^	700	2	Air	–	Multilayer (Solar Absorber)
Ta^[^ ^47^ ^]^	500	2	Vacuum	–	Multilayer (Thermal Absorber)
Mo^[^ ^32^ ^]^	1000	3	Vacuum	–	Metamaterial (Thermal Emitter)
Mo^[^ ^33^ ^]^	1200	24	Ar	–	Nanopillar (Selective Emitter)
Nb^[^ ^48^ ^]^	1000	4	Vacuum	Al_2_O_3_	Nanoantenna
Au^[^ ^49^ ^]^	800	8	Air	Al_2_O_3_	Nanostructure
Ir^[^ ^34^ ^]^	1000	6	Vacuum	HfO_2_	Multilayer (Selective Emitter)
LBSO (This work)	1000	8	Air	–	MIM cavity (Thermal Emitter)

The scanning electron microscope (SEM) in Figure 2c shows the changes in surface morphology after exposure to high temperatures. There is no significant change on the surface when the temperature is increased to 700 °C; however, small particles with radii varying from 10 to 20 nm are generated on the surface that increase in size with increasing temperature of up to 900 °C. At 1100 °C, the formation of randomly distributed rectangular features of a few hundred nanometers was observed on the surface. From the total reflectance at an incident angle of 8° and a diffuse (scattered) reflectance at normal incidence (Figure S5, Supporting Information), we notice that rectangular features formed on the surface at 1100 °C may have an impact on the optical performance, whereas there is no discernible scattering of incident light from the LBSO films exposed to temperatures below 1000 °C.

The out‐of‐plane X‐ray diffraction (XRD) patterns (Figure 2d) display the crystal structure of the LBSO after heating at various temperatures. The as‐grown LBSO film has diffraction peaks only at (00α), referring to a highly crystalline *c*‐axis oriented perovskite crystal structure. Interestingly, we observe a negligible shift of LBSO peak (Δθ_(002)_ ≈0.03°) despite heating at a temperature of 1100 °C in air. Strain characterization of the as‐deposited LBSO film with the reciprocal space map (RSM) in Figure 2e provides a possible explanation for the remarkable thermal stability from the crystal structure of the LBSO film. The diffraction peak position of the as‐grown LBSO film is located on a relaxation line, implying that there is no lattice strain and stress in the LBSO film in the as‐deposited state. In general, notable XRD peak shifts in crystalline films by thermal‐annealing are observed when the crystal lattices of the films are strongly strained in the presence of defects and dislocations.^[^ ^40^ ^]^ Therefore, we expect that the thermal stability of the LBSO film to be a result of the unstrained (fully relaxed) lattice in the as‐deposited state.

**Figure 3:**
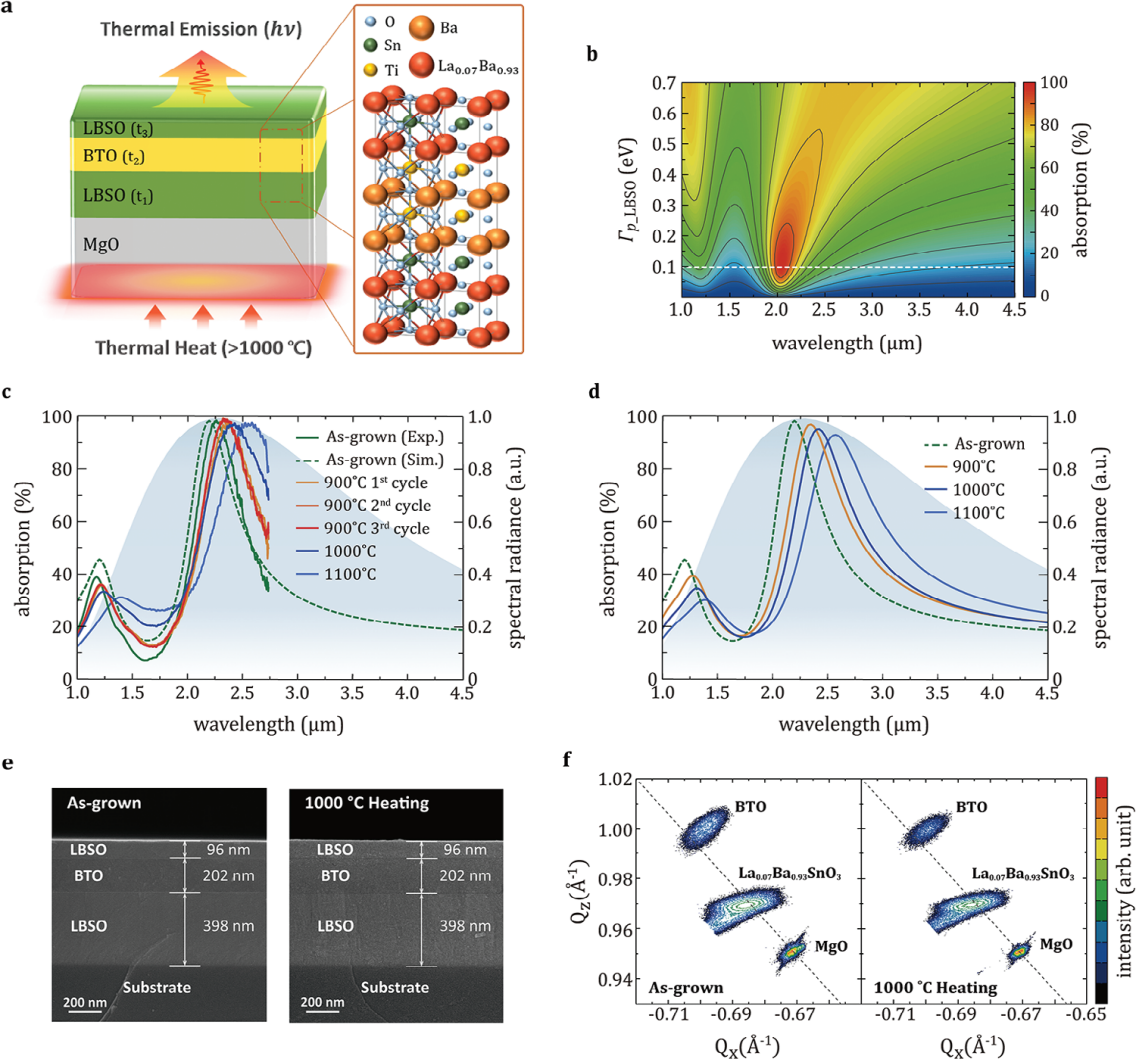
Long‐term thermal stability of refractory LBSO‐based MIM thermal emitter in air atmosphere. a) Schematic image of the refractory thermal emitter design and crystal structure of nanocavity based on LBSO/BTO/LBSO multilayers. b) Simulated absorption spectrum of the thermal emitter as a function of damping coefficient of LBSO thin film ($\Gamma_{p\_\mathrm{LBSO}})$ and wavelength. $\Gamma_{p\_\mathrm{LBSO}}$ of 7 wt.% La‐doped BSO film optimized in this work corresponds to the data represented by the white dashed line. c) Measured and d) simulated absorption spectra of the MIM structure at room temperature and after heating cycles at various high temperatures. A normalized blackbody radiation spectrum obtained at 1000 °C is also shown in the background for comparison. e) SEM cross‐sectional view and f) Reciprocal space mapping (RSM) of the (224) diffraction of MIM thermal emitter before heating and after 1000 °C heating cycle. Gray‐dashed line corresponds to the condition of strain relaxation.

As a next step to verify the applicability of refractory LBSO films to practical thermal processing as well as for TPV systems, we designed a selective narrow‐band thermal emitter in the NIR regime based on an MIM Fabry–Pérot nano‐cavity.^[^ ^20^, ^31^, ^34^, ^35^, ^41^ ^]^ The schematic of the MIM thermal emitter is shown in **Figure** **3**a. Perovskite BTO film was chosen as the dielectric spacer considering the lattice parameter of each layer (lattice constant of LBSO: 4.1 Å and BTO: 4.0 Å) for crystal growth on MgO substrate with minimal lattice strain. In general, nanopatterning of practical refractory metal is required to achieve narrow‐band spectral absorption due to its high optical loss and large magnitude of real permittivity. On the other hand, the low optical loss in optimized LBSO thin films enables a narrow‐band thermal emission with simple MIM geometry without nanopatterning as depicted in the simulated absorption spectrum of MIM thermal emitter by changing Γ_p_ of LBSO film (Figure 3b). We designed the thermal emitter to achieve a near‐unity absorption at a wavelength of 2.2 µm which corresponds to the peak of black body radiation at 1000 °C. The thickness of bottom LBSO layer (*t*_1_), BTO layer (*t*_2_), and top LBSO layer (*t*_3_) was set to 400, 200, and 100 nm, respectively. The details on the design and optimization of MIM thermal emitter are described in Figure S6 (Supporting Information).

Figure 3c shows the measured absorption spectrum of MIM thermal emitter, obtained by UV–vis spectroscopy between 1.0 and 2.7 µm. The measured absorption spectrum of the as‐fabricated thermal emitter is in good agreement with the simulated absorption spectrum based on the optical properties of as‐deposited LBSO film. To examine the long‐term stability of MIM thermal emitters, repeated heating cycles were performed by maintaining the sample at high temperatures in air for 8 h. Similar to changes in the optical properties of LBSO films under high temperature, the narrow band absorption peak of the thermal emitter exhibits a slight shift with increasing temperature of up to 1000 °C, while a notable shift is observed at 1100 °C. After the initial modification of characteristics upon first heating cycle, absorption spectra of thermal emitters were preserved with repeated heating cycles. Figure 3d shows the simulated absorption spectra of the thermal emitter with the optical properties of the LBSO thin film modified after heating at temperatures of 900, 1000, and 1100 °C, respectively, which are well‐matched with the experimental data. Based on the cross‐sectional SEM image and RSM data, we confirm that exposure to high temperatures of up to 1000 °C does not significantly affect to the design and the crystal structure of the LBSO‐based MIM thermal emitter. Therefore, it is feasible to design a thermal emitter tailored to desired operating temperatures by estimating the performance with modified optical properties of the LBSO film upon heating. Lastly, we performed the heating process at 900 °C for 24 h to confirm the long‐term stability, as shown in Figure S7 (Supporting Information). We clearly observed that absorption spectra of thermal emitters were preserved regardless of heating duration.

**Figure 4:**
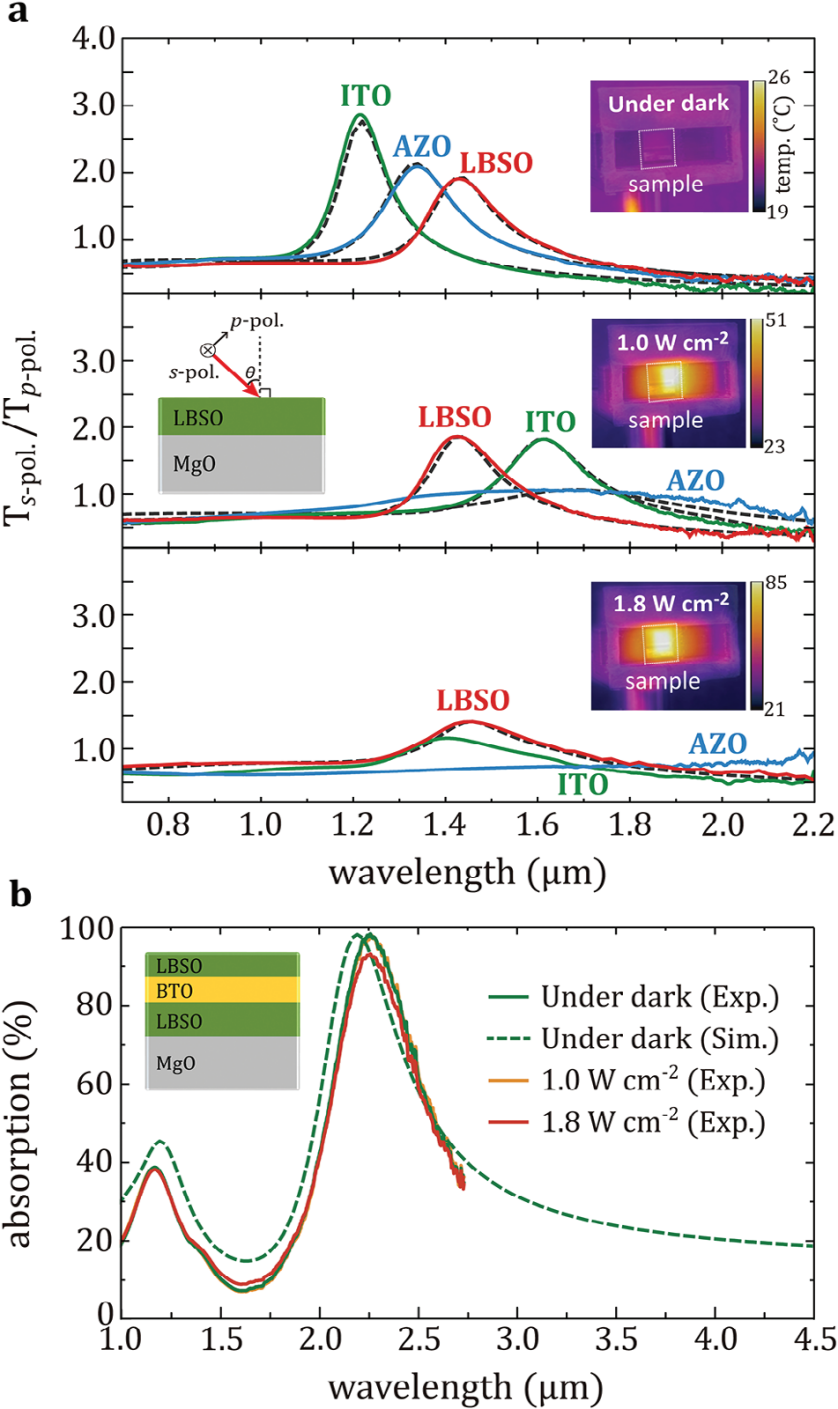
Durability of LBSO film and LBSO‐based MIM thermal emitter to intense laser irradiation. a) The ratio of transmittance for *p*‐polarized (*T*_TM_) and *s*‐polarized (*T*_TE_) with an angle of incidence at 60° under dark and laser illumination with average power of 1.0 and 1.8 W cm^−2^. Inset: Thermal images of LBSO films obtained by IR camera. b) Optical absorption spectra of the MIM emitter under dark and laser illumination with 1.0 and 1.8 W cm^−2^.

In addition to investigating the stability of LBSO film at high temperatures using thermal sources, we evaluated the durability of the NZI property upon laser irradiation because intense optical excitation such as sunlight is one of the main heating sources for emission systems operating at high temperatures in practical applications. For this experiment, we prepared ITO, AZO, and LBSO films with dielectric functions as depicted in Figure 1b. Under UV excimer pulsed laser illumination with an average power of 1.0 W cm^−2^ (peak power of 5 MW cm^−2^) for 30 min, the LBSO film retained its optical properties as shown in **Figure** **4**a. In contrast, the ENZ wavelengths of the AZO and ITO films are significantly red‐shifted with their peaks broadened owing to the increased optical loss. When the average power is increased to 1.8 W cm^−2^ (peak power of 9 MW cm^−2^), AZO and ITO films were significantly damaged, and we are no longer able to analyze these films. Strikingly, the ENZ wavelength of the LBSO is approximately constant despite etching of the films down to 90 nm (see Figure S9, Supporting Information), even though the optical loss is increased due to surface cracks at grain boundaries and thickness reduction. We further performed the same experiment on the MIM thermal emitter. It can be seen that the absorption of LBSO‐based MIM thermal emitter is maintained under the laser excitation with an average power of 1.0 W cm^−2^, but the absorption was slightly decreased when an average power of 1.8 W cm^−2^ was used as shown in Figure 4b. Considering that the laser powers used in this experimental set up is similar to the conditions of excimer laser annealing (ELA), our results suggest that the optical properties of the LBSO materials can be retained under exposure to UV excitation with an intensity that is high enough to induce strong interband absorption causing melting and ablation of the films associated with localized heating.

## 3 Conclusion

In this study, crystalline LBSO thin film exhibits NZI property in the NIR regime similar to conventional metal oxides, and the unstrained lattice structure of LBSO helps to achieve superior stability under thermal heating and intense laser irradiation. Compared to the existing refractory materials summarized in Table 2, it is clear that the LBSO system has the potential as a refractory material for practical applications operating in various gaseous (e.g., oxygen and nitrogen) environments due to its thermal stability in air. For efficient control of thermal emission with NZI materials, it is essential to choose a material with their ENZ bandwidths closer to the desired thermal emission spectrum. Therefore, the tunable optical properties of LBSO through doping can provide the flexibility in designing thermal emitters with tailored operating spectrum. Moreover, with a combination of strong nonlinear efficiency in ENZ property and superior durability against intense laser irradiation, the LBSO system is a great material platform for nonlinear optics including carrier dynamics, harmonic generations, and saturable absorptions. Thus, we expect that the introduction of this new refractory NZI material into the realm of nanophotonics will expand the application domain and enhance the performance of various devices in energy harvesting, high‐temperature material processing, aerospace technologies and other high temperature optical systems.

## 4 Experimental Section

### Sample Preparation

The LBSO films were deposited on MgO substrates with (100) orientation by the PLD using a KrF excimer laser (Coherent, wavelength of 248 nm, energy density of 1.4 J cm^−2^, and repetition rate of 1 Hz). The LBSO target was purchased from Toshima Co, Ltd., and the doping concentration of La was 3, 5, and 7 wt.%. The AZO films were deposited on soda‐lime glass substrates by the same PLD maintaining the deposition temperature at 200 °C with an energy of 1.4 J cm^−2^ in the absence of oxygen.

### Simulation Methods

Numerical simulations of the LBSO‐BTO‐LBSO MIM structure was carried out in the frequency domain by using COMSOL Multiphysics simulation software. The absorption spectra were calculated via BTO layer thickness and bottom LBSO layer thickness to determine the optimized thickness of each layer as shown in Figure S6 (Supporting Information). The measured dielectric properties of LBSO and BTO were used in the simulations.

### Thermal Emitter Fabrication

A BTO layer sandwiched between two LBSO layers was deposited on a MgO substrate to form a LBSO‐BTO‐LBSO MIM structure by PLD. Top LBSO layer (100 nm) and bottom LBSO layer (400 nm) were deposited at 750 °C, with oxygen pressure of 100 mTorr and energy density of 1.4 J cm^−2^, which are the optimized conditions in this work. The BTO layer was deposited at the same temperature and energy density, but with an oxygen pressure of 10 mTorr. The deposition of the entire structure was performed in situ within the PLD chamber.

### High‐Temperature Thermal Treatments

All the films were thermally treated using an electric furnace (CWF 1200, Carbolite) at various temperatures ranging from 200 to 1100 °C. Specifically, AZO and ITO films were exposed to temperatures of up to 500 °C, while the LBSO film was exposed to the full temperature range. The samples were maintained at the peak temperature for 8 h and the furnace was slowly heated and cooled at a rate of 5 °C min^−1^ to minimize film damage.

### Laser Irradiation

For UV laser irradiation, the same KrF excimer laser used for film deposition was employed. The laser frequency was set to 10 Hz and the laser pulse duration was 20 ns. The duration for the laser illumination was 30 min for an average power of 1.0 W cm^−2^, but the laser with an average power of 1.8 W cm^−2^ was illuminated for a relatively short duration (10 min) owing to the etching of the film. During the laser irradiation, the real‐time temperatures of the samples were measured and imaged using a thermal camera (FLIR E8).

### Optical Measurement

The optical properties of all the films before and after heating were characterized by spectroscopic ellipsometry (SE MG‐1000). The dielectric function of the films was retrieved by fitting the Drude model to ellipsometry data. To confirm the extracted dielectric functions, a simulation on transmittance spectra using COMSOL Multiphysics was performed. The linear optical properties of the films were characterized by a UV–vis spectrophotometer (UV‐3600 Plus, Shimadzu, Japan) analysis, with wavelengths ranging from 700 to 2200 nm. The incident beam was polarized by rotating a linear polarizer to measure the *p‐*polarized and *s‐*polarized transmittance.

### XRD and RSM Characterization

Structural analyses of the samples were conducted by using a high resolution X‐ray diffraction (HRXRD, Rigaku ATX‐G) with CuKα. A symmetric Ge[220] monochromator was used on the primary beam with a scan width and scan speed of 0.01° and 0.4° min^−1^, respectively. Reciprocal space mapping (RSM) was performed around the (224) diffraction spots to estimate the extent of relaxation of the deposited thin films.

## Conflict of Interest

The authors declare no conflict of interest.

## Supporting information

Supporting Information

## Data Availability

The data that support the findings of this study are available from the corresponding author upon reasonable request.
