# Supplementary material for: Perovskite Lanthanum‐Doped Barium Stannate: A Refractory Near‐Zero‐Index Material for High‐Temperature Energy Harvesting Systems
Source: Adv Sci (Weinh). 2023 Nov 23;11(2):2302410. doi: 10.1002/advs.202302410 (PMC10787089; doi:10.1002/advs.202302410)
Supplement: Supplementary file 1 — Supporting Information [file ADVS-11-2302410-s001.pdf]

ADVANCED SCIENCE
Open Access

Supporting Information

for *Adv. Sci.*, DOI 10.1002/advs.202302410

Perovskite Lanthanum-Doped Barium Stannate: A Refractory Near-Zero-Index Material for High-Temperature Energy Harvesting Systems

*Hyebi Kim, Geunpil Kim, Young-Uk Jeon, Wonjun Lee, Byeong-Hyeon Lee, In Soo Kim, Kwanil Lee, Soo Jin Kim and Jongbum Kim**

Supporting Information

Perovskite Lanthanum Doped Barium Stannate: A Refractory Near-Zero-Index Material for High-Temperature Energy Harvesting Systems

*Hyebi Kim, Geunpil Kim, Young-Uk Jeon, Wonjun Lee, Byeong-Hyeon Lee, In In Soo Kim, Kwanil Lee, Soo Jin Kim and Jongbum Kim**

Hyebi Kim, Geunpil Kim, Young-Uk Jeon, Wonjun Lee, In Soo Kim, Kwanil Lee, Jongbum Kim
Nanophotonics Research Center, Korea Institute of Science and Technology (KIST), Seoul 02792, Republic of Korea
E-mail: jbkim@kist.re.kr

Hyebi Kim, Geunpil Kim, Young-Uk Jeon, Wonjun Lee, Soo Jin Kim
School of Electrical Engineering, Korea University, Seoul, 02841, Republic of Korea

Byeong-Hyeon Lee
Advanced Analysis Center, Korea Institute of Science and Technology (KIST), Seoul 02792, Republic of Korea

In Soo Kim
KIST-SKKU Carbon-Neutral Research Center, Sungkyunkwan University (SKKU), Suwon 16419, Republic of Korea

1. Energy-dispersive X-ray spectroscopy (EDS) quantitative analysis of $La_xBa_{(1-x)}SnO_3$ with diverse lanthanum(La) doping rate.

a

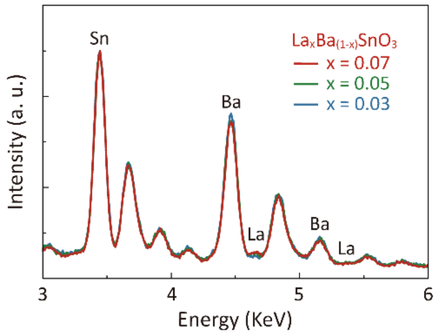

b

Element	x = 0.07	x = 0.05	x = 0.03
La (wt%)	2.23	1.46	0.86
Ba (wt%)	29.80	26.50	26.84
Sn (wt%)	25.55	22.03	22.02
Mg (wt%)	21.12	26.47	26.86
O (wt%)	21.30	23.55	23.41
Lanthanum Doping Ratio (wt%)	6.96	5.22	3.10

Figure S1. a) EDS spectra and b) elemental composition of La-doped BSO thin films with 3, 5, and 7 wt%.

In order to confirm the lanthanum (La) stoichiometry in La-doped BSO thin films fabricated via pulsed laser deposition (PLD), we conducted EDS analysis. We fabricated La-doped BSO thin films with precise doping rates, employing customized stoichiometric PLD targets. Given that the stoichiometry of the deposited films closely matches the target stoichiometry under optimal conditions, the desired lanthanum stoichiometry of 3, 5, and 7 wt% has been successfully realized at 3.10, 5.22, and 6.96 wt%, respectively.

2. ENZ modes of transparent ENZ thin films.

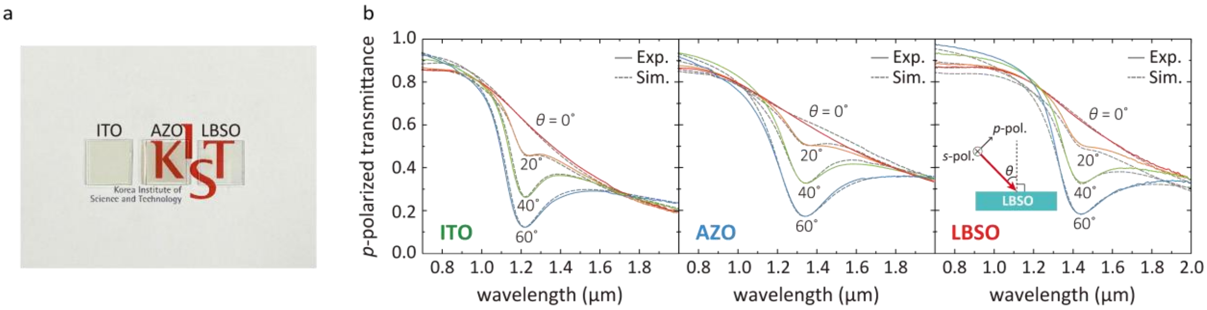

Figure S2. a) Original picture of the transparent ITO, AZO, and LBSO thin films. b) *P*-polarized transmittance of the as-grown ITO, AZO, and LBSO thin film with incidence angles θ=0, 20, 40 and 60°.

Here we demonstrate all oxide-based transparent epsilon-near-zero materials; ITO, AZO, and LBSO. These thin films exhibit high transmittance (>80%) in the visible region. The resonance dips resulting from the excitation of the Ferrell-Berreman mode is clearly observed in the near IR region. Each dip is positioned near its ENZ wavelength; 1.21 μm for ITO, 1.34 μm for AZO and 1.44 μm for $La_{0.07}Ba_{0.93}SnO_3$, as shown in Figure 1b.

3. Surface morphology and ENZ parameter of LBSO thin films grown at various deposition conditions.

The condition of LBSO films grown by pulsed laser deposition (PLD) depends on several deposition parameters including the partial oxygen pressure and the substrate temperature. We fine-tuned these

parameters to achieve low optical loss and ultra-smooth surface by monitoring the surface morphologies and ENZ wavelengths. Specifically, we deposited LBSO films with various oxygen pressures from 50 to 150 mTorr (Figure S3a) and the substrate temperatures from 700 to 800 °C (Figure S3b). Overall, high temperature (> 750°C) and low oxygen partial pressure (< 100 mTorr) cause discontinuities on the film surface. Low temperatures (< 700°C) allow a smooth surface profile, but this condition leads to an increase in the damping coefficient.

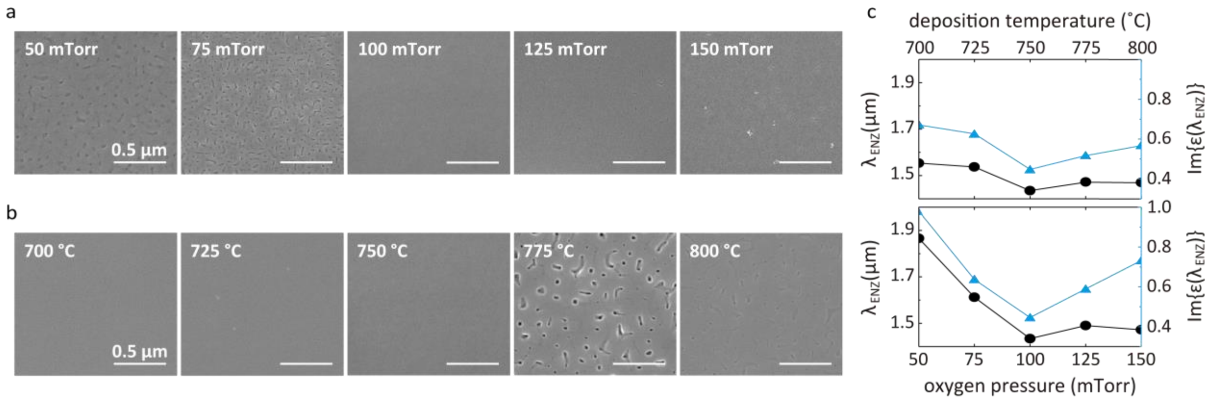

Figure S3. SEM images of LBSO thin films deposited at various a) oxygen pressure and b) substrate temperature. c) ENZ wavelengths (λ_{ENZ}, black line) and imaginary part of permittivity at each ENZ wavelength ($\text{Im}\{\varepsilon(\lambda_{ENZ})\}$, blue line) of the LBSO films grown under the conditions presented in a) and b).

4. Optical transmittance spectra of ENZ thin films under various temperatures in air.

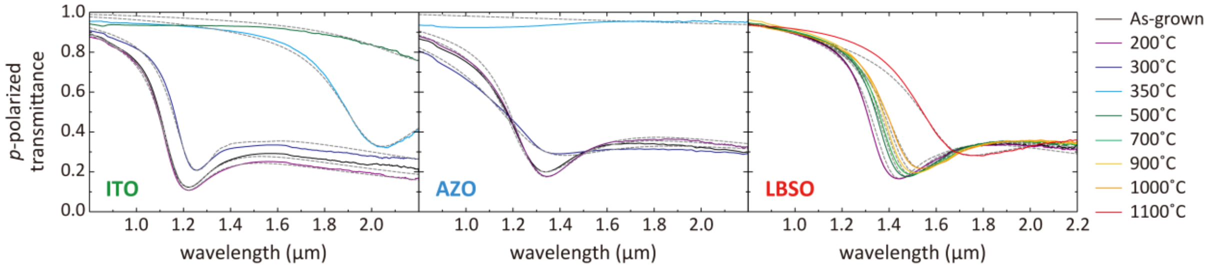

Figure S4. *P*-polarized transmittance of the ITO, AZO, and LBSO films exposed to various temperatures in air, ranging from 200 to 1100 °C. The corresponding temperature range is marked in the legend on the right side of the figure. The measured transmittance spectra of ENZ thin films are in good agreement with the simulated transmittance spectra (grey dashed lines) using the extracted dielectric functions.

As shown in Figure 2, we demonstrated the refractory property of the LBSO thin film through heating processes in air ranging from 200 to 1100 °C. After a cycle of heating and cooling, we characterized the *p*-polarized transmittance with an incident angle of 60° to monitor the change of the ENZ wavelength and optical loss. This was accomplished by observing the spectral location and intensity of the absorption dip, induced by the ENZ mode. Figure S4 shows that the absorption dip of the LBSO films exhibited a slight shift from 1.44 to 1.53 μm with increasing temperature of up to 1000 °C. It is noteworthy that the ENZ wavelengths of the ITO and AZO films underwent significant changes, even when exposed to relatively low temperatures, below 400 °C.

5. Scattered reflectance of rectangular features formed by exposure to high temperature.

As shown in Figure 2c, small particles with radii varying from 10 to 20 nm are generated on the surface that increase in size with increasing temperature of up to 900 °C. However, at 1100 °C, the formation of randomly distributed rectangular features of a few hundred nanometers is observed on the surface.

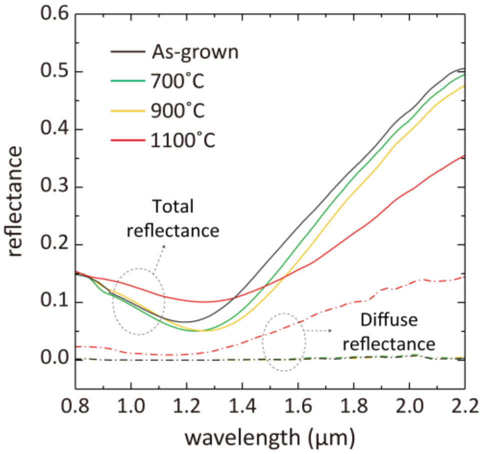

Figure S5. Total (solid lines) and diffuse (dashed lines) reflectance spectrum. There is no diffuse (scattering) of incident light from the LBSO film exposed to temperatures of 700 and 900 °C.

From the total reflectance at an incident angle of 8° and a diffused (scattered) reflectance at normal incidence, we notice that rectangular features formed on the surface may have an impact on the optical performance. Notably, there is no scattering of incident light from the LBSO films exposed to high-temperature approaching 900 °C, whereas the light is strongly scattered from the film exposed to 1100 °C, as the films are reflective in the NIR range.

6. Optimizing Metal-Insulator-Metal (MIM) structure layer thickness through simulation.

We employed COMSOL Multiphysics to calculate the thickness dependent absorption of the thermal emitter based on MIM structure, which is illustrated in Figure 3a. Figure S6 displays the calculated absorption spectra by sweeping a) the BTO film thickness from 0 to 300 nm and b) the bottom LBSO film thickness from 0 to 650 nm. The absorption wavelength can be tuned by adjusting BTO layer thickness because cavity resonance is shifted, allowing the selection of the desired emission wavelength. As seen in b), the thickness of bottom LBSO layer impacts on absorption intensity without changing the emission wavelength. To achieve the near-unity absorption, a thickness of 400 nm was selected for bottom LBSO layer.

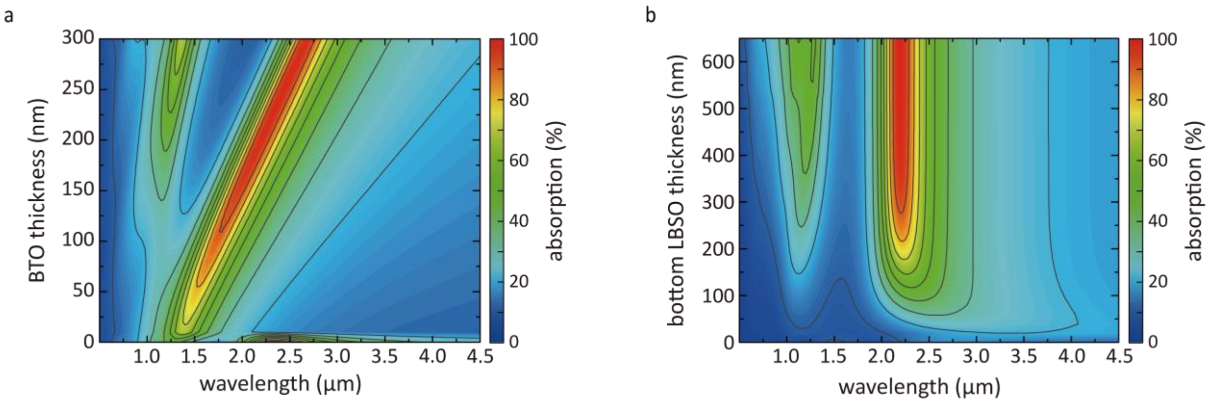

Figure S6. Dispersion maps of calculated absorption spectra as a function of a) the thickness of the BTO layer and b) the thickness of the bottom LBSO layer.

7. Thermal durability of MIM structure for longer durations of heating processes.

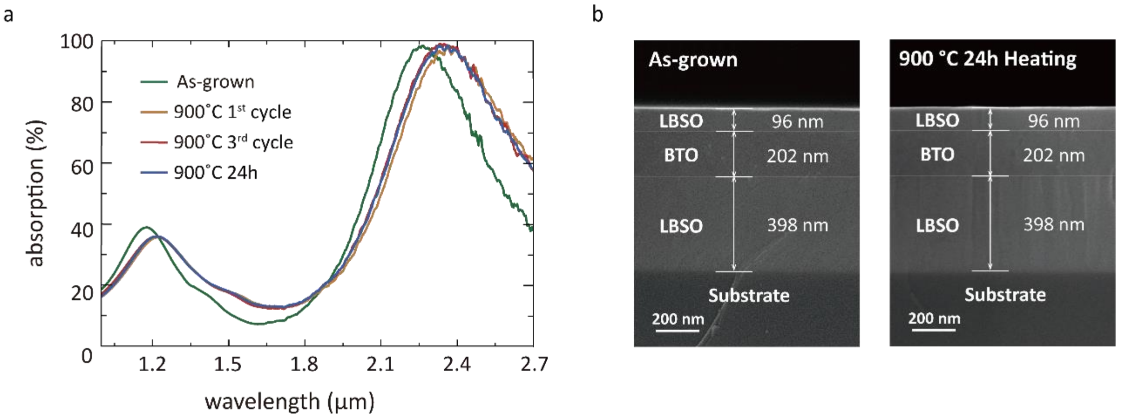

Figure S7. a) Absorption spectra and b) SEM cross-sectional view of the MIM structure at room temperature and after a heating process in ambient air for 24 hours.

8. Morphological changes of ENZ thin films under excimer laser annealing (ELA).

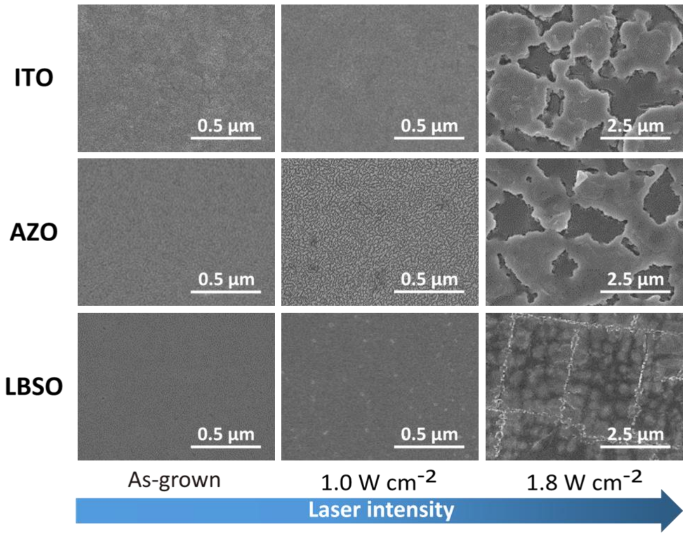

Figure S8. SEM images of the ITO, AZO, and LBSO thin films under dark (as-grown) and after laser irradiation at 1.0 and 1.8 W cm^{-2}

9. Optical transmittance of ENZ thin films under excimer laser annealing (ELA).

The ENZ mode of AZO and ITO can be strongly affected by the laser irradiation with an average power of 1.8 W cm^{-2} as shown in Figure S8. On the other hand, the ENZ wavelength of LBSO film is maintained, whereas the absorption induced by ENZ mode is weaker due to the decrease of film thickness. The reduced film thickness after exposure to excimer laser with the average power of 1.8 W cm^{-2} was confirmed by the ellipsometry measurement.

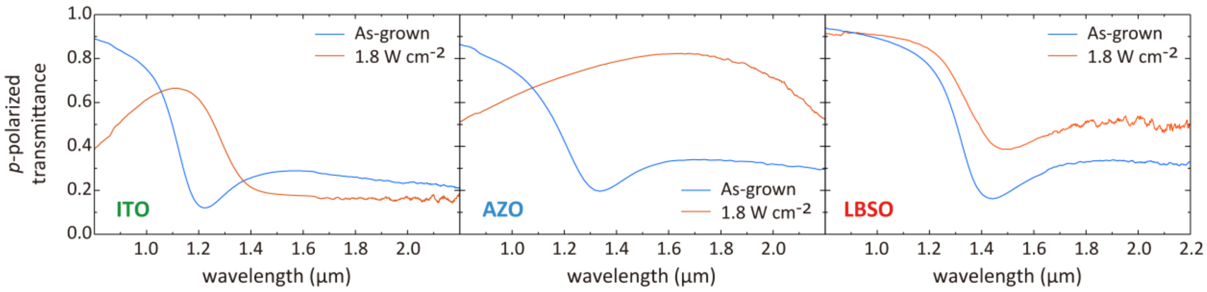

Figure S9. *P*-polarized optical transmittance spectra with an incidence angle of 60° of ENZ thin films under dark (blue) and after laser illumination (orange) at average power of 1.8 W cm^{-2}.
